# Epigenetic and metabolic regulation of epidermal homeostasis

**DOI:** 10.1111/exd.14305

**Published:** 2021-03-10

**Authors:** Roland N. Wagner, Josefina Piñón Hofbauer, Verena Wally, Barbara Kofler, Matthias Schmuth, Laura De Rosa, Michele De Luca, Johann W. Bauer

**Affiliations:** ^1^ Department of Dermatology and Allergology University Hospital of the Paracelsus Medical University Salzburg Austria; ^2^ EB House Austria Research Program for Molecular Therapy of Genodermatoses Department of Dermatology and Allergology University Hospital of the Paracelsus Medical University Salzburg Austria; ^3^ Research Program for Receptor Biochemistry and Tumor Metabolism Department of Pediatrics University Hospital of the Paracelsus Medical University Salzburg Austria; ^4^ Department of Dermatology Medical University Innsbruck Innsbruck Austria; ^5^ Holostem Terapie Avanzate S.r.l. Center for Regenerative Medicine "Stefano Ferrari" Modena Italy; ^6^ Center for Regenerative Medicine "Stefano Ferrari" Department of Life Sciences University of Modena and Reggio Emilia Modena Italy

**Keywords:** epidermal stem cells, epidermolysis bullosa, epigenetics, keratinocytes, miRNAs, mitochondria

## Abstract

Continuous exposure of the skin to environmental, mechanical and chemical stress necessitates constant self‐renewal of the epidermis to maintain its barrier function. This self‐renewal ability is attributed to epidermal stem cells (EPSCs), which are long‐lived, multipotent cells located in the basal layer of the epidermis. Epidermal homeostasis – coordinated proliferation and differentiation of EPSCs – relies on fine‐tuned adaptations in gene expression which in turn are tightly associated with specific epigenetic signatures and metabolic requirements. In this review, we will briefly summarize basic concepts of EPSC biology and epigenetic regulation with relevance to epidermal homeostasis. We will highlight the intricate interplay between mitochondrial energy metabolism and epigenetic events – including miRNA‐mediated mechanisms – and discuss how the loss of epigenetic regulation and epidermal homeostasis manifests in skin disease. Discussion of inherited epidermolysis bullosa (EB) and disorders of cornification will focus on evidence for epigenetic deregulation and failure in epidermal homeostasis, including stem cell exhaustion and signs of premature ageing. We reason that the epigenetic and metabolic component of epidermal homeostasis is significant and warrants close attention. Charting epigenetic and metabolic complexities also represents an important step in the development of future systemic interventions aimed at restoring epidermal homeostasis and ameliorating disease burden in severe skin conditions.

## 1 INTRODUCTION

The skin has a very high cellular turnover rate that, through epigenetic and transcriptional re‐programming, swiftly adapts to environmental stressors such as wounding and barrier disruption. Epidermal homeostasis – the balance between proliferation, differentiation and loss of cells in the stratified epithelium of the skin – sustains tissue integrity and function.^1^, ^2^ Within the skin, epidermal stem cells (EPSCs) reside in the basal layer of the epidermis where they are attached to the basal membrane, which separates the epidermis from the underlying dermis. As stem cells differentiate, they move upward through the different layers of the epidermis towards the surface of the skin.^1^

The rate by which adult epidermal stem cells renew themselves and yield daughter cells depends on developmental stage, external injury, steady‐state tissue turnover and remodelling. Several models of epidermal differentiation and regeneration have been posited to explain the nature and behaviour of EPSCs located within the basal layer of the epidermis.^3^, ^4^ The hierarchical model of epidermal homeostasis proposes the existence of a limited number of slow‐cycling long‐term stem cells within the basal layer that self‐renew and give rise to fast‐cycling transit‐amplifying cells.^5^ According to the stochastic model, on the other hand, all basal cells have equal potential to either divide or directly differentiate.^3^, ^6^ The existence of slow‐ and fast‐cycling stem cells that occupy spatially distinct skin regions and are capable of producing unique differentiated lineages suggests yet another possibility.^7^ Recent data in human 3D cultures suggest that there is a striking variety of signalling processes in the basal layers of the epidermis despite the relatively stable architecture of the terminally differentiated layers.^8^ Which of the different models of stem cell differentiation and regeneration most accurately describes EPSC behaviour *in vivo* is still a subject of ongoing research.^9^, ^10^ Combining cell labelling and linage tracing experiments Piedrafita *et al*. found compelling evidence for the stochastic model of epidermal homeostasis. Their data suggest a state of neutral clonal competition where a population of cells with balanced stochastic cell fate generates, on average, one proliferating and one differentiating daughter cell.^10^ Consistent with earlier reports on grafting experiments in immune compromised mice,^11^ clones develop into widely varying sizes and arise from any point in the basal layer. Importantly, it seems likely that EPSC behaviour in animal models only partially recapitulates the situation observed in the human epidermis. Moreover, deliberate in vivo lineage tracing in humans is not feasible. Nonetheless, recent epidermal grafting studies^12^ provided important mechanistic understanding of epidermal regeneration in humans (discussed below).

Through control of gene expression and homeostasis, aspects of the epigenome regulate almost every biological process, from cellular differentiation and maintenance of phenotypes to onset of disease and ageing.^13^, ^14^ Epigenetic mechanisms such as DNA methylation, histone tail modifications, chromatin accessibility and changes in DNA architecture are tightly correlated with normal cellular function, while their dysregulation manifests in aberrant gene expression and disease.^15^ According to a contemporary definition, epigenomics is defined as “the study of molecules and mechanisms that can perpetuate alternative gene activity states in the context of the same DNA sequence”.^14^ Because of their essential role in establishing specific transcriptional configurations, epigenetic mechanisms govern many aspects of EPSC proliferation, as well as differentiation of their descendants.^16^, ^17^, ^18^, ^19^ Uncharacteristic epigenetic modifications often associate with a loss of transcriptional fidelity, unchecked proliferation, de‐differentiation, and malignant epidermal to mesenchymal transition.^20^ At the same time, pronounced changes in the epigenetic landscape often accompany, and are critical for, resolving challenges to epidermal homeostasis induced by changes in the local microenvironment or external stimuli, such as injury.^21^

Ageing and a variety of diseases, such as chronic inflammation or cancer, manifest themselves in characteristic changes of the epigenetic profile.^22^, ^23^, ^24^ A multitude of other factors, ranging from DNA damage to dietary‐ or drug‐induced metabolic changes, are known to affect the epigenetic status as well. For example, exposure to high altitude,^25^, ^26^ cancer‐associated elevated concentration of lactate,^27^ and increased uptake of dietary methionine^28^, ^29^ have been linked to epigenetic changes and thus highlight the intricate connection between metabolic events and alterations in the epigenome. The pivotal role of mitochondrial energy metabolism in regulating epigenetic events and epidermal homeostasis will be discussed below.

The epigenome is structured into distinct, but interconnected layers ranging from overall chromatin structure and organization to specific histone and DNA modifications. Histones are predominantly modified by methylation, acetylation and phosphorylation, but they can be adapted by other modifications such as ubiquitination, sumoylation, ribosylation and citrullination.^30^ While DNA methylation is the most prominent and studied epigenetic modification (see Box 1|Mechanisms of DNA methylation), other aspects of the epigenome include RNA methylation,^31^ and the expression of coding and non‐coding RNAs, most notable the expression of microRNAs (miRNAs, discussed below).^32^ In recent years, research in epigenomics has been enormously propelled by a multitude of large consortia including the NIH Roadmap Epigenomics Mapping Consortium,^33^, ^34^ International Human Epigenome Consortium,^35^ ENCODE project,^36^, ^37^, ^38^ the Genotype‐Tissue Expression (GTEx) project,^39^, ^40^ the Human Biomolecular Atlas Program (HuBMAP)^41^ or the 4D nucleome project.^42^ The EWAS data hub – comprising normalized DNA methylation array data from 75 K samples – is now available for epigenome‐wide association studies (EWAS).^43^

BOX 1 **Mechanisms of DNA methylation**

DNA methylation is a biochemical process denoted by the addition of a methyl group to cytosines in DNA. Cytosine is methylated at the 5’ position of the pyrimidine ring to form 5‐methylcytosine (5mC). In mammals, DNA methylation almost exclusively occurs in CpG dinucleotides, with the cytosines on both strands being methylated. The human genome contains 56 million CpG sites of which about 70–80% are methylated.^44^ Methylated CpGs are predominantly associated with repetitive elements. Clusters of unmethylated CpG sites – so‐called CpG islands (CGIs) – are associated with promoter and enhancer regions. Importantly, most cell types display relatively stable DNA methylation patterns and dynamic regulation occurs for only about 20% of autosomal CpGs.^45^ These CpGs participate in the genomic regulation of key lineage‐specific factors. Cell‐, tissue‐ and condition‐specific differences in methylation define so‐called differentially methylated regions (DMR).^44^ Hypermethylation generally refers to an increase in methylation and can be found in regions where most cytosines are methylated, like in heterochromatin. Hypomethylation denotes a loss of methylation. Regions where most cytosines are non‐methylated are found in euchromatin and active gene promoters. Cancers generally exhibit global hypomethylation, whereas regional hypermethylation in the promoter regions of tumour suppressor genes is frequently observed.

Methyl groups are added to cytosines by DNA methyltransferase (DNMTs). DNMT1 predominantly methylates hemimethylated CpGs and therefore is crucial for maintaining methylation during DNA replication.^46^ Although DNMT1 displays a very high fidelity, there is an inevitable global loss of methylation with each cell division. DNMT3a and DNMT3b are de novo methyltransferases that can methylate both unmethylated and hemimethylated DNA, and orchestrate the establishment of DNA methylation patterns early in development.^47^ Conversely, TET (Ten eleven translocation) enzymes actively remove methyl groups from DNA by oxidation with the production of 5‐hydroxymethylation as an intermediate.^48^

## 2 EPIDERMAL HOMEOSTASIS IN HEALTH AND DISEASE

### 2.1 Epigenetic regulation of epidermal homeostasis

**FIGURE 1:**
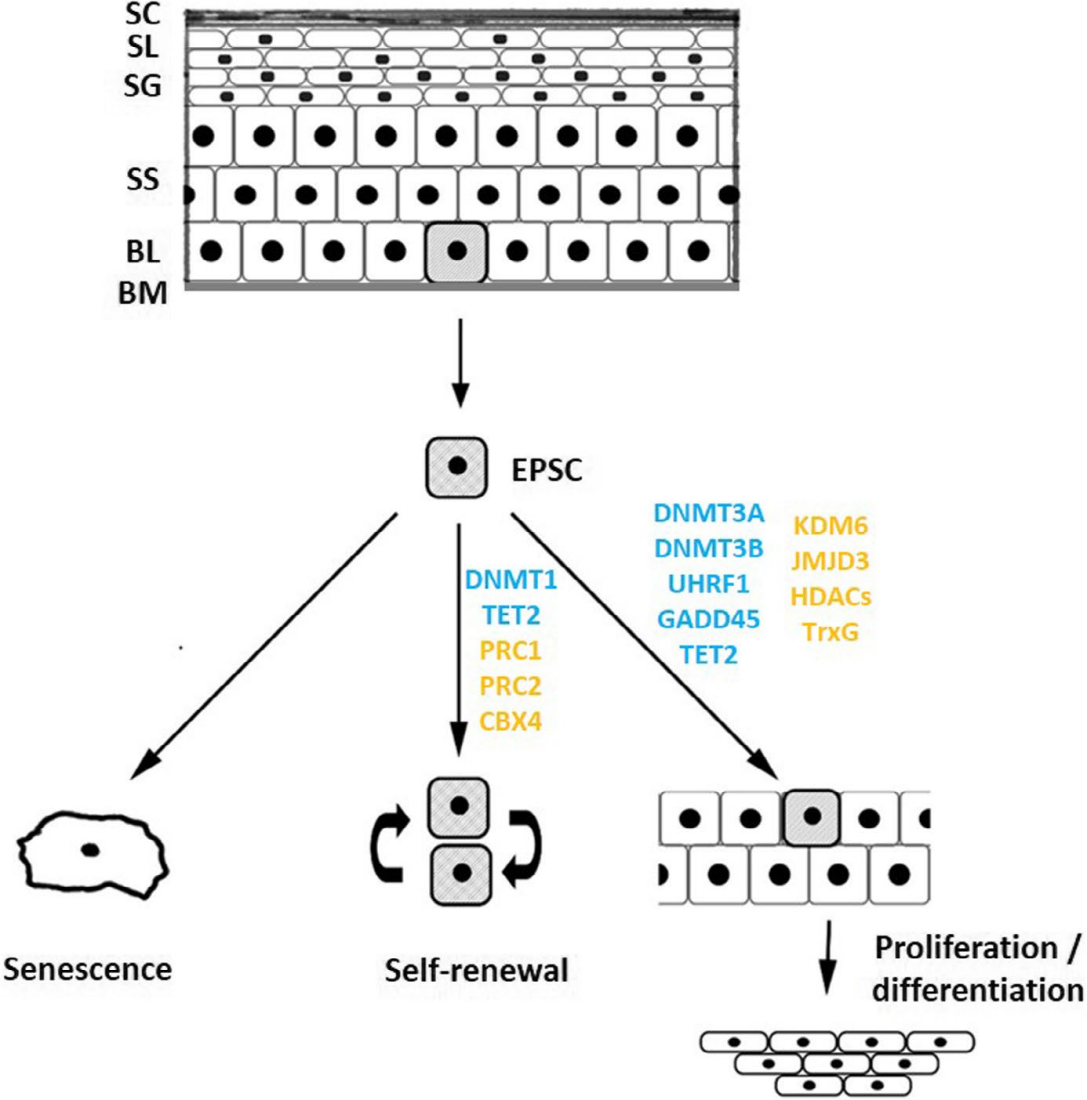
Epigenetic effectors and epidermal homeostasis. Maintenance and differentiation of EPSCs critically governs epidermal homeostasis. Individual, proliferating EPSCs (indicated as shaded cells) are located in the basal layer. As cells differentiate, they progressively move upward through the various layers of the epidermis. Eventually, these cells lose their nuclei before forming the layers of the outermost stratum corneum. Multiple epigenetic effectors regulate EPSC self‐renewal, proliferation and differentiation. These factors control DNA methylation (indicated in blue), histone modification and chromatin remodelling (indicated in orange). Abbreviations: BL, basal layer; BM, basement membrane; DNMT, DNA methyltransferase; EPSC, epidermal stem cell; HDAC, histone deacetylases; PRC, polycomb repressive complex; SC, stratum corneum; SG, stratum granulosum; SL, stratum lucidum; SS, stratum spinosum; TET, ten‐eleven translocation; TrxG, trithorax group proteins

There is ample evidence that epigenetic mechanisms, such as DNA methylation, histone modifications or changes in DNA topology, contribute to epidermal homeostasis and differentiation^49^, ^50^, ^51^, ^52^ (summarized in Figure 1 and Table 1). Epigenetic regulation has also been analysed in wound healing and functional links between chromatin architecture and gene expression in keratinocytes have been found.^53^, ^54^, ^55^, ^56^

**TABLE 1:** Epigenetic factors in skin homeostasis

Epigenetic effector	Main activity	Major phenotypes of loss of function	Reference
DNA modifiers			
DNMT1	Maintains methylation of CpGs (‘maintenance DNMT’)	Defects in EPSC maintenance and proliferation; disrupted epidermal stratification and hair follicle development; development of alopecia	46, 153, 154, 155
DNMT3A	De novo methylation of CpGs (‘de novo DNMT’)	Defects in EPSC differentiation; Cutaneous tumourigenesis; squamous transformation; skin ageing	59, 60, 156, 157
DNMT3B	De novo methylation of CpGs (‘de novo DNMT’)	Defects in EPSC differentiation; squamous transformation; skin ageing	59, 60, 157
TET1	Demethylation of CpGs	Dysregulated EPSC kinetics	158
TET2	Demethylation of CpGs	Dysregulated EPSC kinetics; defects in EPSC proliferation and migration; Skin ageing	59, 157, 158, 159
UHRF1	Co‐factor, binds hemi‐methylated DNA and recruits DNMT1	Defects in epidermal differentiation	46, 160
Gadd45A/B	Co‐factor, involved in DNA demethylation	Defects in epidermal differentiation	46
Histone modifiers			
KDM6B	H3K27 demethylase	Epidermal differentiation	161
JMJD3	H3K27 demethylase	Delayed wound healing	162
Histone demethylases	hypomethylation of histone H3K4/9/27me3	impaired epithelial cell differentiation	50
HDAC1/2	Suppression of gene expression	Decrease SC proliferation, impaired stratification, alopecia	163, 164
Trichostatin‐A	HDAC inhibitor	HFSC, IFE proliferation, block of terminal differentiation	165, 166
Chromatin remodelers			
PRC1	Suppression of gene expression	Defects in EPSC differentiation	167, 168, 169, 170
PRC2	Suppression of gene expression	Defects in EPSC differentiation	167, 171
BMI1	Component of PRC1, mediates monoubiquitination of H2AK119	EPSC maintenance and proliferation	172
CBX4	Component of PRC1, mediates monoubiquitination of H2AK119	EPSC maintenance and proliferation	168
EZH1	Component of PRC2, catalyzes methylation of H3 K27	EPSC maintenance and proliferation	173
EZH2	Component of PRC2, catalyzes methylation of H3 K9 and H3 K27	EPSC maintenance and proliferation	167, 173
SUZ12	Component of PRC2, catalyzes methylation of H3 K9 and H3 K27	EPSC maintenance	171
TrxG	Activation of gene expression	Defects in EPSC differentiation	174

Abreviations: DNMT, DNA methyltransferase; HDAC, Histone deacetylases; PRC, polycomb repressive complex; TET, Ten‐eleven translocation; TrxG, trithorax group proteins

Disrupted chromatin regulation, prompted by the loss of PRC1, results in impaired epidermal tissue integrity and blistering skin resembling human skin fragility syndromes.^49^ In regards to histone modifications, chemical inhibition of histone demethylases impairs differentiation of inter‐follicular stem cells and delays injury repair.^50^ Chronic sun exposure is associated with distinct histone acetylation changes and altered gene expression in human photodamaged skin.^57^ Histone acetyltransferase (HAT) activity is dependent on zinc and depletion of zinc results in decreased HAT activity. The epithelial zinc transporter ZIP10 epigenetically regulates human epidermal homeostasis by modulating zinc availability and histone acetyltransferase activity.^58^ Reduced ZIP10 activity or depletion of zinc leads to reduced HAT activity and decreased expression of genes, such as filaggrin or metallothionein, involved in epidermal homeostasis.^58^

Likewise, dynamic epigenetic regulation of DNA methylation (see Box 1|Mechanisms of DNA methylation) is critical for the maintenance of EPSC status and proliferative capacity. A progressive loss of DNA methylation patterns caused by forced depletion of DNMT1 in the epidermis leads to failure of EPSC self‐renewal and tissue regeneration.^46^ Consistently, *DNMT1* expression is normally restricted to the basal layers of the epidermis containing the EPSC population, and mostly absent in the outer differentiated layers. The de novo DNMTs, DNMT3A and DNMT3B, also critically contribute to EPSC homeostasis by controlling enhancer methylation and active chromatin conformation of stem cell relevant genes.^59^ Specifically, co‐localization of DNMT3A and TET‐2 at target enhancers results in 5‐hmC formation and gene activation.^59^ Interestingly, DNMT3A and DNMT3B also seem to protect the epidermis from tumourigenesis since the loss of these genes in the mouse epidermis promotes squamous transformation.^60^ In atopic dermatitis, DNA methylation patterns from patients differ significantly from those of healthy controls.^61^, ^62^, ^63^ Moreover, epigenetic dysregulation caused by diminished TET‐1 and TET‐2 expression and concomitant reduction of 5‐hmC marks leads to unbalanced EPSCs proliferation and maturation in psoriasis.^64^ Although the cause of diminished TET expression in psoriasis remains unresolved, reconstitution of TET expression increases 5‐hmC levels and results in normalized EPSCs kinetics.

The P16ink4/Rb signalling pathway further highlights the critical role of epigenetic regulation in maintaining epidermal homeostasis. P16ink4 is a potent inhibitor of the G1/S phase transition and therefore a tumour suppressor gene and entry point to cellular senescence. P16ink4 is also crucial for controlling EPSC behaviour and is in turn an important target of multiple epigenetic regulatory processes involving DNMTs, TET enzymes, Polycomb group proteins and Jumanji protein families.^65^ Interestingly, epigenetic drift or disrupted epigenetic regulation, respectively, have also been linked to loss of epidermal homeostasis in skin ageing and rare skin conditions (see Box 2|Epigenetic drift and skin ageing and Box 3|Epigenetic regulation in disorders of cornification).

BOX 2 **Epigenetic drift and skin ageing**

The observation that global DNA methylation marks stochastically change with age, led to the idea that the methylation status of a distinctive and – compared to the entire methylome – narrow set of CpG sites could be used to predict the chronological and biological age of an organism. Thus, global assessment of age‐related DNA methylation changes can be used to configure so‐called epigenetic clocks for highly accurate age prediction. Since the first development of a DNA methylation age estimator, the predictive power of epigenetic clocks has been constantly improving and contemporary epigenetic clocks are considered the most accurate biomarkers of ageing available. In skin ageing, methylation data have been used to predict the chronological age of sample donors with high accuracy. One of the most recent iterations of an epigenetic clock, the Skin&Bood clock, is based on assessing the methylation status of 391 CpG sites and predicts the chronological age of subjects from human fibroblasts, keratinocytes, buccal cells, whole skin, blood and saliva samples with high precision.

Interestingly, accelerated epigenetic ageing is observed in disease and cancer. A hallmark of ageing is the increased cell‐to‐cell variability in epigenetic marks and gene expression. This epigenetic drift invariably leads to a decline in stem cell number and function and entails the onset of age‐associated illnesses. Changes in methylation variability were accompanied by reduced connectivity of transcriptional networks. These findings thus define the loss of epigenetic regulatory fidelity as a key feature of the ageing epigenome.^66^

BOX 3 **Epigenetic regulation in disorders of cornification**

Dominant‐negative mutations in *KRT9* cause diffuse palmoplantar keratoderma (PPK), a debilitating genodermatosis for which there is no effective treatment.^67^ The disease phenotype of PPK is limited to palmoplantar surfaces where KRT9 protein is expressed, while there is little KRT9 expression in other body locations. Notably, previous work has shown that the site‐specific Homeobox protein Hox‐A13 (*HOXA13*) in fibroblasts can be modulated by Aza‐C, a DNA methylation inhibitor, and implicated the presence of *HOXA13*‐expressing fibroblasts in palmoplantar skin to be important for site‐specific *KRT9* expression via Wnt family member 5A (*WNT5A*) in these body locations.^68^

Ichthyosis vulgaris (IV), characterized by *generalized* dry skin and scaling, is the most common monogenic genodermatosis. It is caused by mutations in the profilaggrin/filaggrin gene (*FLG*). It is well‐known that IV families also have a high incidence of atopic dermatitis (AD), a common inflammatory skin disease with often severe itching and association with hay fever and asthma. However, it remains unknown why some family members in IV families develop both, IV and AD, and others display IV only. One study reported a lack of correlation between methylation in the *FLG* gene promoter and allergic phenotypes.^69^ Conversely, DNA methylation within the *FLG* gene, specifically within the CpG site ‘cg07548383’ was reported to significantly interact with *FLG* sequence variants on the risk for eczema,^70^ although this study did not provide direct evidence of DNA methylation modulating *FLG* expression. Furthermore, another genome‐wide study revealed differences in DNA‐methylation in lesional AD as compared to healthy control skin.^63^ In this publication, differences in DNA‐methylation are described for genes that are involved in regulating epidermal homeostasis and innate immunity, i.e. *KRT6A OAS2*, *S100A* and *LRRC8C*, the latter with expression probes in *trans* with CD36. In turn, CD36 was shown to be increased in states of skin barrier disruptions^71^ and mutations in CD36 cause ichthyosis prematurity syndrome^72^ with skin barrier abnormalities and disturbances in epidermal lipid metabolism.^73^ In contrast, the level of demethylation of *FOX3i1* in circulating regulatory T cells (Tregs) is similar between AD and control subjects.^74^ However, the demethylation of the *FCER1G* promoter in monocytes^75^ and the *TSLP* promoter in keratinocytes showed differences.^76^ Thus, current knowledge implies epigenetic regulation of epidermal homeostasis in AD and may account for the association between IV and AD.

### 2.2 The role of miRNAs in epidermal homeostasis

The overall contribution of miRNAs to skin homeostasis was demonstrated in functional studies in which conditional epidermal knockout of key elements of the miRNA processing machinery in murine embryos, namely Dicer^77^, ^78^ and Dgcr8,^79^ resulted in a severe skin phenotype, characterized by follicular dysplasia, epidermal hyperproliferation, and defects in barrier function, accompanied by a failure to thrive and early postnatal lethality. Numerous miRNAs have now been assigned specific roles in skin morphogenesis, homeostasis and tissue regeneration (as reviewed in^80^, ^81^, ^82^). Under normal physiological circumstances, miRNAs are predicted to mediate the post‐transcriptional control of up to 60% of all expressed genes.^32^ Additionally, they are intricately interconnected in epigenetic networks. Their expression can be affected by the classic epigenetic modifications of promoter DNA methylation and histone acetylation, and they themselves can control the epigenetic machinery by directly targeting individual enzymatic components.^83^, ^84^ Moreover, reports of miRNAs co‐localizing to specific promoter regions as components of different DNA‐binding complexes indicate potentially active roles in chromatin remodelling.^84^ This places miRNAs at the core of epigenetic/miRNA regulatory circuits that can significantly impact a plethora of cell functions.^85^

MiRNome profiling, coupled with functional validation of candidates, continues to drive our understanding of miRNA regulation of prominent skin processes, both in health and disease contexts (see Table 2). In the context of epidermolysis bullosa (EB) – a rare genetic disorder of the skin discussed in more detail below – the role of miRNAs in disease pathogenesis is beginning to surface. To date, three miRNAs have been described to modulate EB‐associated complications such as fibrosis (miR‐29b,^86^, ^87^ miR‐145^88^, ^89^) and cancer (miR‐10b^90^). The repetitive destabilization of the extracellular matrix that accompanies recessive dystrophic EB (RDEB) upon injury results in progressive soft tissue fibrosis with debilitating consequences, such as tumour development.^91^ miR‐145‐5p was shown to be upregulated in RDEB‐fibroblasts, which typically exhibit more contractile features than their wild type counterparts, indicating a potential correlation between RDEB severity and miR‐145‐5p levels, by contributing to skin fibrosis.^88^ Indeed, inhibition of miR‐145‐5p resulted in a downregulation of α‐SMA, TAGLN and JAG1, all of which are contractile markers, leading to a reduction of fibrotic traits.^88^ Another miRNA, miR‐29, which directly targets the disease‐causing gene COL7A1, as well as the essential COL7A1 expression regulator SP1, was found to be downregulated in RDEB fibroblasts.^87^ Furthermore, in a complex network, TGF‐ß was shown to be a further activator of *COL7A1* expression and at the same time reduces miR‐29 levels via SMAD phosphorylation.^87^, ^92^, ^93^ Apart from *COL7A1* regulation, miR‐29 family members were also shown to influence DNA methylation by targeting distinct DNA methyl transferases^94^ and proteins involved in DNA demethylation.^95^

**TABLE 2:** Selected microRNAs with function in skin biology

MicroRNA	Function	Reference
Homeostasis and morphogenesis		
miR‐34a	Induces differentiation. Anti‐proliferative function	175
miR‐34c	Suppresses differentiation, involved in senescence	176
miR‐125b	Represses stem cell differentiation and promotes stem cell renewal	177
miR‐184	KC differentiation	178
miR‐203	Repressor of p63. Regulator of keratinocyte differentiation	179
miR‐205	Enhances migration	180
miR‐210	Pro‐angiogenic. Cell‐cycle regulation. Hypoxa‐miR	181
Epigenetic targets		
miR‐29 family	DNMT3A‐B, indirectly DNMT1	182
miR‐145	HDAC2	183
miR‐200b	PCGF4	184
miR‐200c	PCGF4	185
miR‐221	HDAC6	186
Cancer		
miR‐10b	Confers of stemness features to tumour cells in cSCCs	90
miR‐21	OncomiR, anti‐apoptotic, pro‐survival	187, 188
miR‐27a	Targets EGFR	189
miR‐34a	Tumour‐suppressor miRNA, targets HMGB1	190
Ageing		
miR‐146a	Involved in fibroblast senescence via regulation of Smad4	191
miR‐181a,b	Involved in senescence in keratinocytes and fibroblasts	192, 193
Fibrosis		
miR‐21	Fibroblast proliferation and transdifferentiation	194
miR‐29b	Regulator of collagen expression	86, 87
miR‐145	Regulation of myofibroblast differentiation	88, 89

Abbreviations: HDAC, Histone deacetylase; PCGF4, Polycomb group RING finger protein 4.

Patients suffering from RDEB are particularly prone to developing exceptionally aggressive squamous cell carcinomas (SCCs). In this context, overexpression of miR‐10b has been attributed a role in conferring stemness to tumour cells, specifically by increasing cell adhesion in 2D and 3D functional models. While miR‐10b is the first miRNA described to be associated with RDEB‐SCCs,^90^ the role of miRNAs in tumourigenesis is generally well‐accepted, and has been described for several tumour entities, among them cutaneous SCCs, affecting diverse mechanisms like migration and proliferation.^96^, ^97^, ^98^

### 2.3 Mitochondrial control of epidermal homeostasis

Emerging evidence suggests that mitochondria are vital regulators of skin physiology.^99^ Epidermal progenitor/stem cells do not rely on the mitochondrial respiratory chain, but still require a functional dynamic mitochondrial compartment.^100^ One main task of keratinocytes is corneocyte renewal and production of stratum corneum‐specific proteins and lipids needed for a functional skin barrier. These processes require high amounts of energy, which is normally generated by oxidative phosphorylation (OXPHOS). During differentiation of keratinocytes in the skin, mitochondrial membrane potential declines and mitochondria undergo phenotypic changes in an apoptosis‐like process.^101^, ^102^, ^103^ A decline in mitochondrial energy production in favour of glycolysis might contribute to the production of lactate in the stratum corneum. Lactate production of keratinocytes is important to skin barrier function as well as the maintenance of skin flexibility.^104^

Mitochondria are the major intracellular source of reactive oxygen species (ROS), predominantly generated via complex I and III of the OXPHOS system.^105^ ROS can inflict oxidative damage on biomolecules, resulting in loss of catalytic and/or structural integrity. With ageing, ROS‐damaged proteins accumulate and OXPHOS activity declines. Accordingly, mitochondrial oxidative stress limits epidermal cell proliferation and stem cell numbers, leading to reduced wound healing in older mice. Interestingly, in young mice, mitochondrial oxidative stress actually accelerates wound healing.^106^ Both naive and differentiating progenitor stem cells (PSCs) activate OXPHOS, whereas primed PSCs rely on glycolysis.

Several mitochondriopathies are associated with skin manifestations, including hair abnormalities, rashes, pigmentation disorders, hypertrichosis and acrocyanosis.^99^ Cytochrome oxidase (complex IV of OXPHOS) activity is greatly reduced in allergic contact dermatitis and ichthyosis, indicating diminished aerobic respiration.^107^ Mutations in the plectin 1 (*PLEC1*) gene cause epidermolysis bullosa simplex (EBS) with muscular dystrophy (EBS‐MD).^108^ PLEC1B, which localizes in the outer mitochondrial membrane, helps to maintain organelle shape and network formation by tethering mitochondria to intermediate filaments.^109^ PLEC1‐deficient cells show a disorganized intermediate filament network and severe mitochondrial dysfunction.^110^, ^111^ Furthermore, in keratinocytes of patients with EBS caused by a mutation of keratin (*KRT*) *5* or *KRT14*, abnormal mitochondrial distribution has been reported.^112^

Interpreting the crosstalk between the nuclear epigenome and mitochondria in both normal physiological function and different diseases is an advancing research topic.^113^, ^114^, ^115^, ^116^ The field distinguishes between anterograde (nucleus to mitochondria) and retrograde (mitochondria to nucleus) signalling. Both communicate intracellular requirements or a need to compensate for a dysfunction to maintain epidermal homeostasis. Pertaining to anterograde signalling, growing evidence suggests that nuclear regulators, including transcription factors, DNMTs and TET demethylases, as well as non‐coding RNAs may be exported from the nucleus and directly impact transcription of the mitochondrial genome.^116^ Moreover, a recently discovered link between toxin‐induced promoter hypomethylation and mitochondrial biogenesis in skin cancer development further highlights the importance of coordinated epigenetic regulation and mitochondrial function.^117^ When it comes to retrograde signalling, an increasing number of studies have identified potential alterations in the epigenetic landscape of the nuclear genome as a consequence of mitochondrial dysfunction.^114^ For example, depletion of mtDNA results in significant changes in methylation pattern of a number of genes. The methylation changes are reversed by the restoration of mtDNA.^118^ In addition, numerous mitochondrially‐derived metabolites serve as regulators or substrates for epigenetic marks, e.g. S‐adenosylmethionine (SAM) is required as a substrate for methylation of many histone proteins but also DNA‐methylation.^119^

### 2.4 Epidermal homeostasis in epidermolysis bullosa

Recently, our understanding of EPSC biology and epidermal homeostasis has been fuelled by advances in treating rare skin conditions such as inherited epidermolysis bullosa (EB), caused by loss of adhesion and cohesion of the skin. EB manifests itself as a wide spectrum of clinically heterogeneous phenotypes. The type of mutated gene, position and nature of the mutation within the respective gene, as well as mode of inheritance, predict the particular subtype of EB.^120^ Phenotypic variability amongst patients with the same or similar mutations, however, remains often unexplained. Frequently, the same mutation results in intra‐ and interfamilial disease variability. Siblings with the same mutation in *COL7A1*, for example, can present with different clinical phenotypes. Even monozygotic twins can show pronounced phenotypic variation for diverse traits, including disease susceptibility and progression. In other rare diseases, discordant phenotypes between monozygotic twins can often be attributed to different epigenetic states and aberrant epigenetic regulation.^121^ Epigenetic modifications in EB remain underexplored at large and inference of the importance of epigenetic mechanisms in influencing disease progression is largely circumstantial. For now, the only published disease modifiers in EB include genes associated with TGF‐β pathway inhibition^122^ and members of the matrix metalloproteinase family (MMP‐1),^123^, ^124^ although the importance of MMP‐1 as a modifier gene remains unclear.^125^, ^126^ In a mouse model of junctional EB (JEB), featuring a hypomorphic mutation in Lamc2, Col17a1 acts as a strong disease modifier.^127^

Through the application of combined *ex vivo* cell and gene therapy, almost the entire epidermis of an EB patient can be reconstituted by genetically corrected long‐lived EPSCs.^12^, ^128^, ^129^ In a series of therapeutic skin transplantations, we discovered that, apart from technical issues, the outcome of the procedure depends on the anatomical site of the initial biopsy, the age of the patient, the genes involved, and, perhaps more importantly, on the microenvironment characterizing the receiving wound bed. The contribution of age is common knowledge in the field and has also been observed by us^128^ in the case of a 49‐year‐old patient vs. a 7‐year‐old patient.^12^ The intrinsic ageing processes of the skin have been revealed to depend on cytoskeletal proteins (e.g. keratins; cytoskeletal proteins including desmosomes, microtubules and microfilaments)^130^ and other cellular processes, like cell cycle control, inflammatory response, signalling and metabolism.^131^, ^132^, ^133^ Moreover, EB per se is a disease not only of skin attachment, but it also displays an ageing phenotype exemplified by a specific gene expression signature.^134^

In the case of JEB, this has been related to dysregulation of the YAP/TAZ pathway, which causes progressive, age‐related depletion of stem cells.^135^ We provided evidence that the reduction of clonogenic potential and the loss of stem cells in primary JEB keratinocytes is associated with perturbation of the YAP/TAZ signalling which renders ex vivo gene therapy cumbersome.^135^ The Hippo signalling pathway, better known for its function in organ size control through its effectors Yes‐associated protein (YAP) and WW domain‐containing transcription regulator 1 (commonly listed as TAZ), has been demonstrated to play a pivotal role in regulating tissue homeostasis and regeneration in skin.^135^, ^136^, ^137^, ^138^, ^139^ The transcriptional regulators YAP and TAZ localize to the nucleus in the basal layer of human and mouse epidermis^135^, ^139^, ^140^ and are elevated during wound healing.^136^, ^137^, ^138^, ^139^ Skin specific deletion of both YAP and TAZ in adult mice leads to hair loss and impairs regeneration after wounding.^136^ YAP expression correlates with stem cell content and it has been reported that nuclear YAP progressively declines with age and correlates with the proliferative potential of epidermal progenitors.^135^, ^139^

**FIGURE 2:**
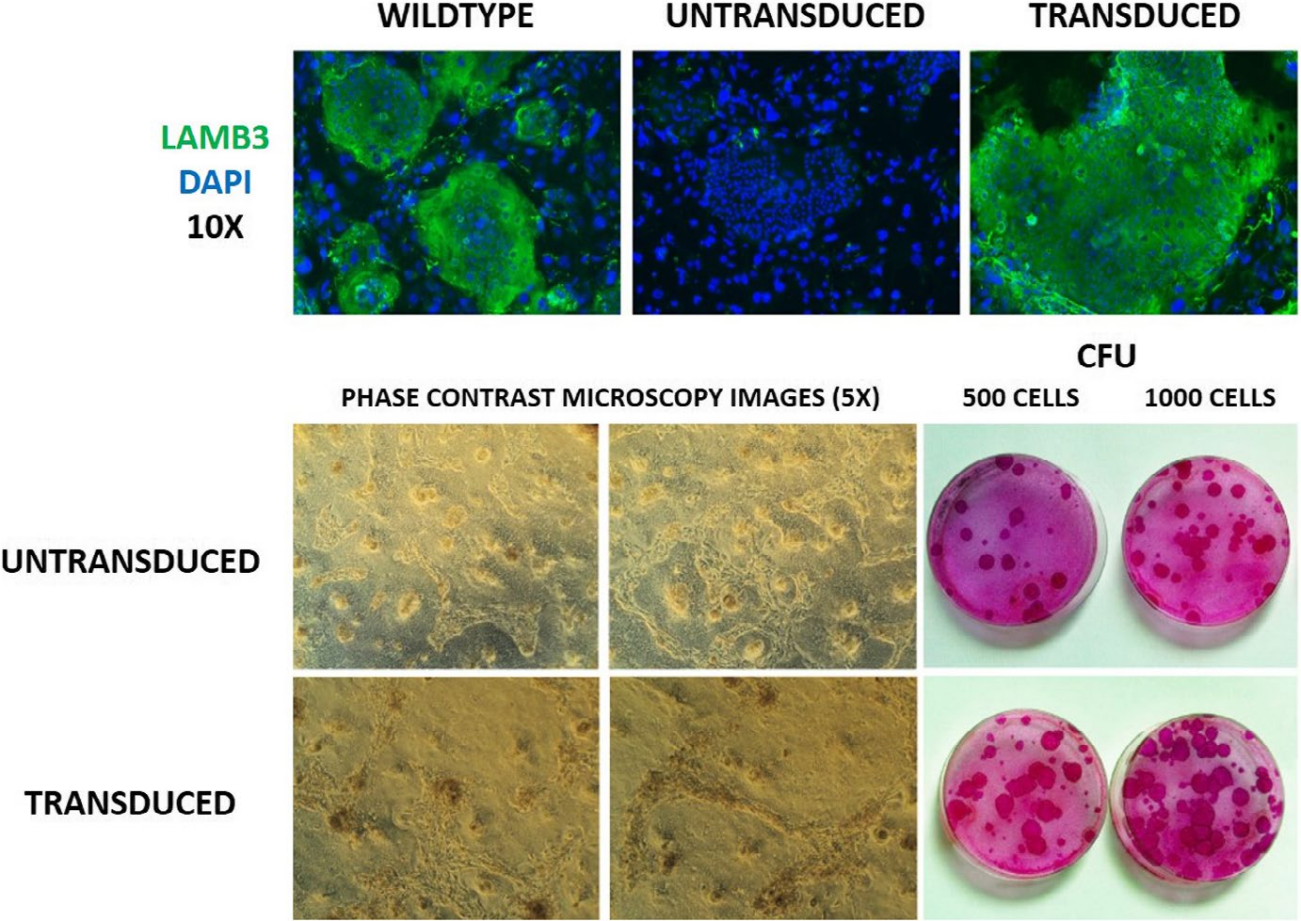
Proliferation potential of primary keratinocytes from EB patients. Keratinocytes from a 49‐year‐old JEB patient were transfected to re‐express LAMB3 protein. Upper panel: LAMB3‐deficient patient keratinocytes in cell culture. After transfection, there is improved clonal potency as can be seen by the increased number and size of red‐stained clones in the lower panel (M. De Luca et al, unpublished results). CFE: Colony‐forming units

Compared to those derived from healthy donors, EPSCs from EB patients are often difficult to culture *ex vivo*. Repeated wounding and sustained proliferative stress may contribute to decreased plasticity and increased exhaustion of EPSCs in EB patients. There are distinct differences in clonogenic ability and proliferation potential in LAMB3‐ and COL7‐deficient keratinocytes. In LAMB3‐deficient keratinocytes, both properties are severely altered, but they can be rescued by transduction with a LAMB3‐expressing vector (Figure 2). This does not hold true for *COL7*‐ or *COL17*‐deficient keratinocytes, which have a proliferative potential similar to that of normal keratinocytes. Therefore, competition between untransduced vs. transduced patient keratinocytes might occur in transplanted areas of dystrophic EB patients, hampering full therapeutic success (M De Luca, JW Bauer, unpublished observation). The cell‐ and molecular‐biological reasons for this constellation have been only partially elucidated.^135^

Most likely, characteristic transcriptional and epigenetic anomalies beyond the causal EB mutation promote the observed differences in proliferative capacities of EPSCs. In general, proper interaction of EPSCs with the basement membrane assures their maintenance and propagation. *LAMA3*, *LAMB3* and *LAMC2* encode subunits of laminin‐332, which is crucial for anchoring epithelial cells to the basement membrane. Reduced or absent expression of functional laminin‐332, caused by mutations in the corresponding genes, accounts for the majority of cases with JEB. Beyond its structural role, Laminin‐332 influences EPSC differentiation and its absence in JEB leads to stem cell depletion.^12^, ^128^, ^135^, ^141^ Aberrant laminin‐332 expression has also been connected to tumour progression. *LAMB3* features a CpG poor promoter region. How the methylation status of non‐CpG island promoters affects gene expression is generally not well defined. Correspondingly, the influence of *LAMB3* promoter methylation on gene expression is somewhat ambiguous. Epigenetic silencing of the LAMB3 gene has been linked to certain cancers^142^, ^143^, ^144^ and resistance to cisplatin treatment.^145^ A different study, however, found promoter hypomethylation and up‐regulated expression of LAMB3 in gastric cancer.^146^ To our knowledge, however, there are currently no published reports on prospective epigenetic differences of EPSCs from EB patients and age‐matched healthy donors.

In light of high somatic mutation rates, stem cell competition further appears to be an important factor in maintaining tissue homeostasis by keeping propagation of stem cell clones with cancer‐causing mutations and abnormal cellular behaviour in check.^147^ At the same time, stem cell competition may also account for the phenomenon of revertant phenotypes in JEB caused by inherited mutations in COL17A1,^148^, ^149^, ^150^ and ichthyosis with confetti caused by mutations in *KRT1* or *10*.^151^, ^152^

## 3 CONCLUSION

The contribution of epigenetic, miRNA‐mediated and mitochondrial events during epidermal homeostasis in health and disease is profound. Substantial progress has been made in understanding the underlying molecular details. Even so and despite the success of progressive treatment options, like *ex vivo* stem cell/‐gene therapy, our understanding of several aspects of basic skin biology is still incomplete. It is clear that the regulation of self‐renewal and proliferative potential of EPSCs is strongly determined by their genetic background, epigenetic signatures, metabolic state and the tissue microenvironment. The combination of these factors may impinge on the clinical outcome of advanced stem cell therapies. Since genetic material is often integrated into the genome of the patient during the procedure, it should be feasible to include supplemental components for manipulating these factors and thus improve the chances for therapeutic success. At the same time, continuing research and a deeper mechanistic understanding of skin homeostasis will likely reveal novel avenues for therapeutics and regenerative medicine in the field of genodermatoses.

## CONFLICT OF INTEREST

The authors declare no conflict of interest.

## AUTHOR CONTRIBUTION

RNW, JWB conceptualized the manuscript. RNW, JPH, VW, BK, MS, LDR, MDL, JWB performed literature review and wrote different paragraphs. All authors read and approved the final manuscript.
